# Normal radiological anatomy of thyroid cartilage in 600 Chinese individuals: implications for anterior cervical spine surgery

**DOI:** 10.1186/s13018-018-0728-y

**Published:** 2018-02-08

**Authors:** Ying-zhao Yan, Chong-an Huang, Qi Jiang, Yi Yang, Jian Lin, Ke Wang, Xiao-bin Li, Hai-hua Zheng, Xiang-yang Wang

**Affiliations:** 1 Department of Spine Surgery, The Second Affiliated Hospital and Yuying Children’s Hospital of Wenzhou Medical University, Second Medical School of Wenzhou Medical University, Zhejiang Spine Surgery Centre, Wenzhou, Zhejiang 325027 China; 2 Department of Orthopaedic Surgery, The Fifth Affiliated Hospital of Wenzhou Medical University, Lishui Central Hospital, Lishui, Zhejiang 323000 China; 3 Department of Internal Medicine, The Sixth Affiliated Hospital of Wenzhou Medical University, The People’s Hospital of Lishui, Lishui, Zhejiang 323000 China; 4 The Second Affiliated Hospital and Yuying Children’s Hospital of Wenzhou Medical University, 109 Xueyuan Xi Road, Wenzhou, Zhejiang 325027 China

**Keywords:** Thyroid cartilage, Cricoid cartilage, Anterior cervical spinal surgery, Anatomy, Positioning landmark, Pharyngoesophageal wall

## Abstract

**Background:**

Thyroid cartilage is an important barrier in anterior cervical approach surgery. The objective of this study is to establish normative values for thyroid cartilage at three planes and to determine their significance on preoperative positioning and intraoperative traction in surgery via the anterior cervical approach.

**Methods:**

Neck CT scans were collected from 600 healthy adults who did not meet any of the exclusion criteria. Transverse diameters (D1, D2, and D3) of the superior border of the thyroid cartilage (SBTC), inferior border of the thyroid cartilage (IBTC), and the trachea transverse diameters of the inferior border of the cricoid cartilage (IBCC) were measured on a horizontal plane.

**Results:**

All measured variables had intra-class correlation coefficients (ICCs) of ≥ 0.7. The differences in transverse diameters on the same plane between males and females were significantly different (all *p <* 0.001). The SBTC is most often at C4 in women (59.5%) and C4/5 in men (36.4%), the IBTC is most often at C5 in women (48.1%) and men (46.2%), and the IBCC is primarily located at C6 in women (45.2%) and C6 or C6/7 in men (34.4%) (all *p* < 0.001).

**Conclusion:**

We present normative values for thyroid cartilage at three planes of SBTC, IBTC, and IBCC in Chinese individuals. The individual and gender differences in the location of the thyroid cartilage and the size of the thyroid cartilage and the cricoid cartilage provide an anatomical basis to localize the skin incision, to predict the difficulty of intraoperative exposure and retractor pulling, and to identify that the thyroid cartilage protected the pharyngoesophageal wall.

## Background

Anterior cervical (AC) surgery is associated with numerous and frequent complications that can be attributed to inaccurate positioning, inadequate exposure, and the use of excessive traction [1–3]. Attempts have been made to reduce the risk of complications in recent years. This has generally been achieved by improving body-surface positioning [1] and by evaluating the impact of the thyroid cartilage on intraoperative retraction [4, 5], as well as exploring hypopharynx/esophagus protective mechanisms.

Preoperative positioning in anterior cervical approach surgery generally utilizes anatomical structures such as the angle of the mandible, hyoid bone, thyroid cartilage, cricoid cartilage, and carotid artery nodule [1, 6]. For example, approximately 70% of the mandibular angle is located at the interval between C2 and C3 [1], while the majority of cervical decompression surgeries are located below the C4 level [7]. Therefore, the angle of the mandible is not suitable for the majority of positions in anterior cervical approach surgery. The thyroid cartilage is closely associated with the cervical vertebra in terms of anatomy. It is located at the C4–C5 level, serves as a prominent anatomical landmark, and is therefore convenient for pre-surgical positioning in the anterior cervical approach.

Additionally, to expose the vertical vertebral level for surgery, significant retraction pressure may be exerted on the thyroid cartilage and posterior pharyngeal wall or esophagus. Wire retractor tension should thus be increased during surgery due to the great thyroid cartilage transverse diameter [4]. However, Han et al. [5] found that tension outside the thyroid cartilage would not increase the intra-esophageal pressure. Additionally, the posterior border of thyroid cartilage (PBTC) at the time of traction could protect the pharyngoesophageal (PE) wall [4]. However, such a standpoint requires further support from thyroid cartilage related-anatomical data.

Few studies in the literature have summarized the distribution of the transverse diameter of the superior and inferior border planes of the thyroid cartilage in addition to the relative cervical vertebra level [8, 9]. This study aimed to measure the neck computed tomography (CT) images of healthy adults to obtain thyroid cartilage or cricoid cartilage imaging parameters to assist in preoperative positioning, to predict the difficulty of intraoperative exposure and retractor pulling, and to verify that the thyroid cartilage protected the PE wall of the anterior cervical approach.

## Methods

### Subject enrollment and data collection

This study received institutional review board approval and followed the principles of the Declaration of Helsinki. From January 2010 to September 2017, a total of 600 adults with healthy outpatient physical examination results at our hospital were enrolled for thyroid cartilage imaging measurements. Patients with no complaints of neck pain were included if they did not meet any of the exclusion criteria. The exclusion criteria were as follows: (1) diseases such as deformity, tuberculosis, or tumor of the larynx, thyroid cartilage, or thyroid, (2) diseases such as congenital deformity, torticollis, and neck stiffness in the neck structure, (3) spinal trauma, infection, tumor, or coronal deformities (Cobb angle > 10°) [10], (4) a history of prior spine surgery, (5) complaints of neck pain that affected activities of daily living, (6) degenerative or pathologic condition of the spine that necessitated physician intervention, (7) non-ambulatory patients, (8) pregnancy, and (9) inconsistent CT scan plane and measurement plane.

Patient height, weight and BMI were recorded after enrollment. All subjects underwent a C1–T1 thin-layer CT scan (layer thickness of 0.50 or 0.75 mm) in our hospital.

### Radiographic analysis

**Fig. 1:**
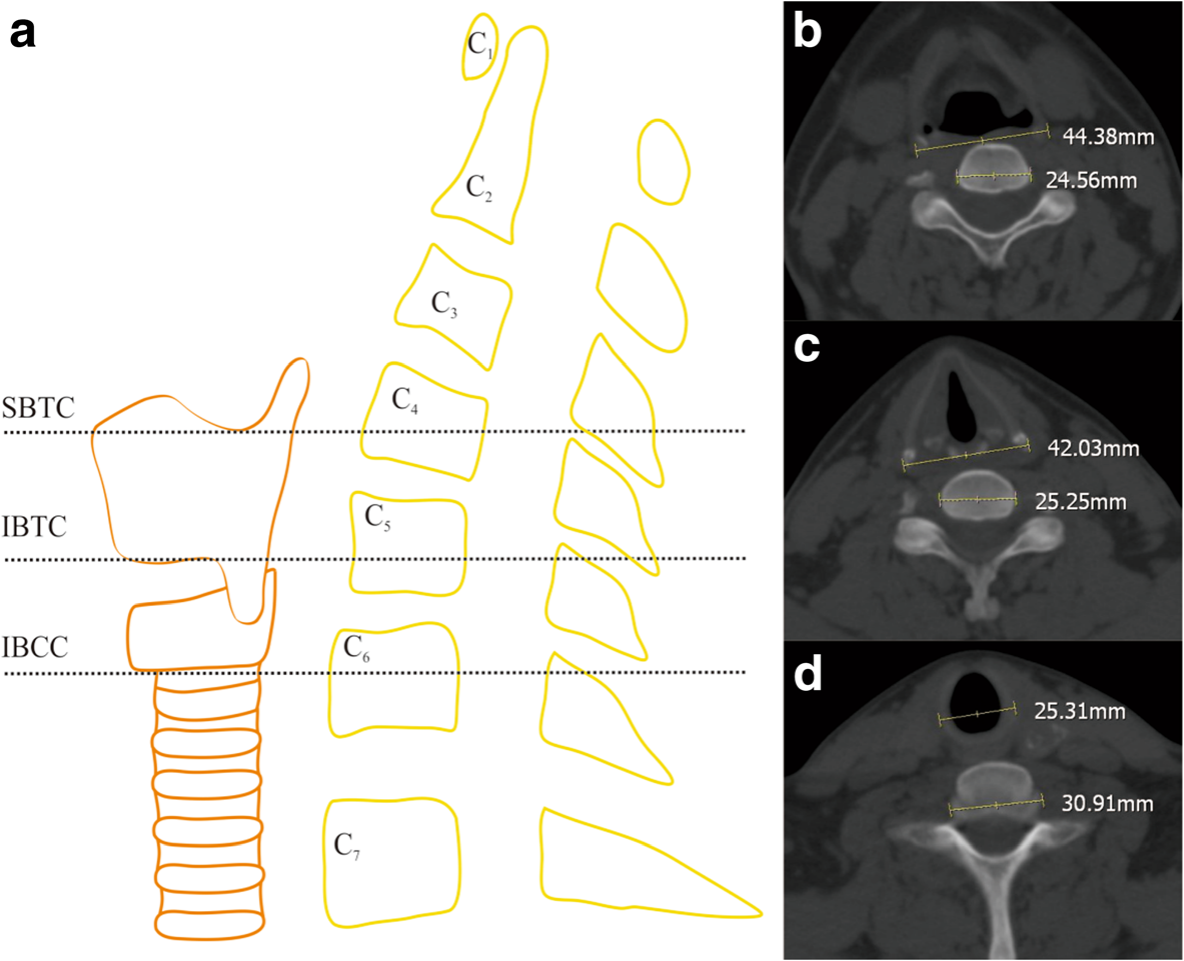
The measurements used for this study. **a** Levels at which the thyroid and cricoid cartilage and cervical vertebrae were measured include the superior border of the thyroid cartilage (SBTC), the inferior border of the thyroid cartilage (IBTC), and the inferior border of the cricoids cartilage (IBCC). **b** The measurement of the SBTC. **c** The measurement of the IBTC. **d** The measurement of the IBCC

The thyroid cartilage imaging parameters of 600 healthy subjects were measured via a picture archiving and communication system with an accuracy of 0.01 mm under the bone window on the thin-layer CT cross section. The following radiographic parameters were measured: transverse diameters of the superior border of the thyroid cartilage (D1), transverse diameters of the inferior border of the thyroid cartilage (D2), and trachea transverse diameters of the inferior border of the cricoid cartilage (D3), as well as the transverse diameters of the corresponding vertebral body and intervertebral disc (V1, V2, and V3). Examples of D1, D2, D3, V1, V2, and V3 are shown in Fig. 1. The number of corresponding vertebral bodies or intervertebral discs on all planes was recorded.

The radiographs were reviewed by two authors who had independently measured more than several hundred cervical CT images prior to this review. The average of both reviewers’ measurements was used for analysis.

### Statistical analyses

The SPSS 19.0 statistical software package (SPSS Inc., Chicago, USA) was used for statistical analyses. Descriptive statistics including the mean, standard deviation, minimum, maximum, and percentiles were calculated for all variables. Measurement data conforming to a normal distribution were expressed as the mean ± standard deviation.

The transverse diameters of the anatomical measurement data in all planes were compared between males and females using independent samples *t* tests. The corresponding cervical level in all measurement planes was expressed as frequency and percentage and was compared using a chi-squared test between males and females. *p* values of less than 0.05 were considered significant.

A Pearson product-moment correlation coefficients table was then constructed to determine the correlations between all radiographic variables and age, weight, height, and BMI. Intra-class correlation coefficients (ICCs) and the kappa statistic were used for continuous and categorical intra-rater reliability data, respectively [9]. The internal consistency of the measurements was characterized as excellent (≥ 0.9), good (≥ 0.7 and < 0.9), acceptable (≥ 0.6 and < 0.7), poor (≥ 0.5 and < 0.6), or unpredictable (< 0.5) [10].

## Results

### Patient characteristics

There were 600 healthy study subjects aged 54.1 ± 10.0 years (range 24 to 77), with a height of 165.0 ± 7.2 cm (range 145 to 180), weight of 63.3 ± 10.1 kg (range 41 to 90), and BMI of 23.1 ± 2.8 kg/m^2^ (range 16.9 to 31.8). Overall, 390 subjects were males aged 24 to 77 years, with a height of 145 to 180 cm, weight of 49 to 90 kg, and BMI of 18.4 to 31.1 kg/m^2^. A total of 210 subjects were females aged 31 to 74 years, with a height of 148 to 173 cm, weight of 41 to 81.5 kg, and BMI of 16.9 to 31.8 kg/m^2^.

Because we intended to establish normative values based on gender, effort was made to maintain an even distribution of age and BMI between males and females. The demographic data are included in Table 1. The differences in age and BMI between males and females were not significant (*p* > 0.05).

**Table 1:** Demographic data of healthy adults

	Total	Male	Female	*p* value
Number of cases	600	390	210	–
Age (years)	54.1 ± 10.0	53.7 ± 9.4	55.1 ± 10.9	0.106
Weight (kg)	63.3 ± 10.1	66.4 ± 9.1	57.5 ± 9.1	0.000
Height (cm)	165.0 ± 7.2	168.6 ± 5.2	158.4 ± 5.6	0.000
BMI (kg/m^2^)	23.1 ± 2.8	23.3 ± 2.6	22.9 ± 3.0	0.069

Data presented as the mean ± SD

The significant difference was compared between males and females

### Radiographic results

Overall, we had good (ICC or Kappa ≥ 0.7) to excellent inter-observer reliability for all included radiographic measurements (Table 2); the majority of measurements exhibited excellent reliability.

**Table 2:** Measurement reliability statistics for various radiographic values

	Kappa	ICC	Inter-rater reliability
CLCEP	0.9		Excellent
Transverse diameter of thyroid cartilage of SBTC (D1)		0.8	Good
Transverse diameter of vertebral body or intervertebral disc of SBTC (V1)		0.8	Good
Transverse diameter of thyroid cartilage of IBTC (D2)		0.9	Excellent
Transverse diameter of vertebral body or intervertebral disc of IBTC (V2)		0.7	Good
Transverse diameter of trachea of IBCC (D3)		0.9	Excellent
Transverse diameter of vertebral body or intervertebral disc of IBCC (V3)		0.9	Excellent

*CLCEP* cervical level corresponding to each plane, *SBTC* plane of superior border of the thyroid cartilage, *IBTC* plane of inferior border of the thyroid cartilage, *IBCC* plane of inferior border of the cricoid cartilage, *ICC* intra-class correlation coefficient

The average values for each variable, including the mean, standard deviation range, and percentiles, are reported in Table 3. The average values by gender are reported in Table 4. The transverse diameters of the thyroid cartilage or trachea (D1, D2, and D3) gradually decreased in all the planes from top to bottom, while those of the vertebral body or intervertebral disc (V1, V2, and V3) gradually increased. Differences on the same plane between males and females were significant (all *p* < 0.001).

**Table 3:** Average values and percentiles for all measured radiographic parameters

	Mean (mm)	Min (mm)	Max (mm)	Percentiles				
				5	25	50	75	95
D1	44.4 ± 5.0	34.0	60.0	36.1	41.1	44.0	47.3	53.2
D2	41.5 ± 4.6	31.2	54.5	34.3	38.3	41.3	45.1	49.4
D3	26.5 ± 3.8	18.8	34.5	20.4	23.2	27.1	29.3	32.6
V1	27.5 ± 2.6	21.4	35.5	23.1	25.9	27.4	29.0	31.9
V2	29.1 ± 3.2	16.2	40.0	24.8	27.1	28.5	30.4	35.9
V3	31.6 ± 3.4	24.1	41.0	26.4	29.1	31.6	33.7	37.3

**Table 4:** Radiographic parameters by gender

	Male (mm)	Female (mm)	*p* value
D1	46.6 ± 4.5	40.4 ± 2.8	0.000
D2	43.7 ± 3.7	37.3 ± 2.8	0.000
D3	28.7 ± 2.5	22.4 ± 1.7	0.000
V1	28.4 ± 2.5	26.0 ± 2.1	0.000
V2	30.0 ± 3.2	27.3 ± 2.1	0.000
V3	32.9 ± 3.0	29.0 ± 2.4	0.000

Data presented as the mean ± SD

*D1*, *D2*, and *D3* transverse diameter of thyroid cartilage or trachea of SBTC, IBTC, and IBCC

*V1*, *V2*, and *V3* transverse diameter of vertebral body or intervertebral disc of SBTC, IBTC, and IBCC

When examining the change in alignment with age, we found that V1 (*r* = 0.124, *p* < 0.05), V2 (*r* = 0.202, *p* < 0.01), and V3 (*r* = 0.130, *p* < 0.01) all increased with age (Table 5). There was no correlation between age and D1, D2, and D3. However, a higher BMI was correlated to an increase in D1 (*r* = 0.148, *p* < 0.01), D2 (*r* = 0.136, *p* < 0.01), and D3 (*r* = 0.215, *p* < 0.01). Interestingly, increasing weight and height were correlated to increasing D1, D2, D3, and V1, V2, and V3 (all *p* < 0.01). These correlations are detailed in Table 5.

**Table 5:** Correlations of all radiographic parameters with age, weight, height, and BMI

	Age	Weight	Height	BMI
D1	−.047	.400^a^	.527^a^	.148^a^
V1	.124^b^	.244^a^	.350^a^	.073
D2	−.073	.394^a^	.531^a^	.136^a^
V2	.202^a^	.163^a^	.331^a^	−.025
D3	.019	.505^a^	.626^a^	.215^a^
V3	.130^a^	.187^a^	.430^a^	−.064

*D1*, *D2*, and *D3* transverse diameter of thyroid cartilage or trachea of SBTC, IBTC, and IBCC

*V1*, *V2*, and *V3* transverse diameter of vertebral body or intervertebral disc of SBTC, IBTC, and IBCC

^a^Correlation is significant at the 0.01 level (2-tailed)

^b^Correlation is significant at the 0.05 level (2-tailed)

**Fig. 2:**
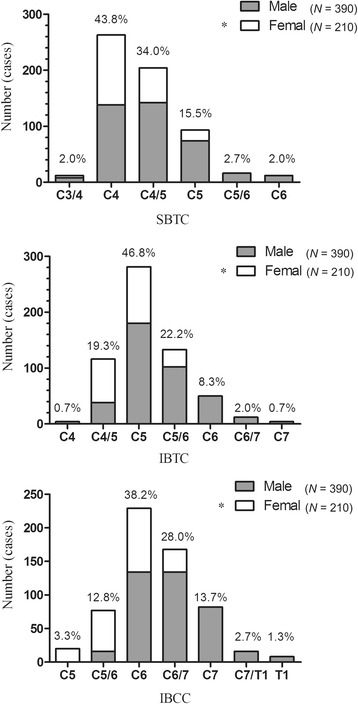
The frequency distribution of the cervical vertebral level corresponding to SBTC, IBTC, and IBCC by gender. *SBTC* superior border of the thyroid cartilage, *IBTC* inferior border of the thyroid cartilage, *IBCC* inferior border of the cricoids cartilage. ^*^p < 0.001, compared with males

The differences in the distribution of the corresponding cervical levels on SBTC, IBTC, and IBCC are reported in Fig. 2. The distribution was also significant between males and females (*p* < 0.001, Table 6). The SBTC corresponded to levels C3/4 to C6 and was mainly located at C4 (*n* = 263), accounting for 43.8% of cases, while 560 (93.3%) were located at level C4 to C5. The IBTC corresponded to levels C4 to C7, with the majority located at C5 (*n* = 281), accounting for 46.8% of cases. Meanwhile, 530 (88.3%) were located at levels C4/5 to C5/6. The IBCC corresponded to levels C5 to T1, with most located at C6 (*n* = 229), accounting for 38.2% of cases. A total of 556 (92.7%) cases were located at levels C5/6 to C7.

**Table 6:** The frequency distribution of cervical segments corresponding to each plane in 600 healthy subjects

Level	SBTC			IBTC			IBCC		
	Male^*^ (%)	Female (%)	All (%)	Male^*^ (%)	Female (%)	All (%)	Male^*^ (%)	Female (%)	All (%)
C3/4	8 (2.1)	4 (1.9)	12 (2.0)	0	0	0	0	0	0
C4	138 (35.4)	125 (59.5)	263 (43.8)	4 (1.0)	0	4 (0.7)	0	0	0
C4/5	142 (36.4)	62 (29.5)	204 (34.0)	38 (9.7)	78 (37.1)	116 (19.3)	0	0	0
C5	74 (19.0)	19 (9.0)	93 (15.5)	180 (46.2)	101 (48.1)	281 (46.8)	0	20 (9.5)	20 (3.3)
C5/6	16 (4.1)	0	16 (2.7)	102 (26.2)	31 (14.8)	133 (22.2)	16 (4.1)	61 (29.0)	77 (12.8)
C6	12 (3.1)	0	12 (2.0)	50 (12.8)	0	50 (8.3)	134 (34.4)	95 (45.2)	229 (38.2)
C6/7	0	0	0	12 (3.1)	0	12 (2.0)	134 (34.4)	34 (16.2)	168 (28.0)
C7	0	0	0	4 (1.0)	0	4 (0.7)	82 (21.0)	0	82 (13.7)
C7/T1	0	0	0	0	0	0	16 (4.1)	0	16 (2.7)
T1	0	0	0	0	0	0	8 (2.1)	0	8 (1.3)
Total	390 (100)	210 (100)	600 (100)	390 (100)	210 (100)	600 (100)	390 (100)	210 (100)	600 (100)

Percentages are shown in brackets

*SBTC* superior border of the thyroid cartilage, *IBTC* inferior border of the thyroid cartilage, *IBCC* inferior border of the cricoid cartilage

^*^p < 0.001 for males versus females using the chi-squared test

## Discussion

In this study, we have presented normative values for D1, D2, and D3 as well as V1, V2, and V3. To the best of our knowledge, these values have not been reported in any previous studies. In addition, we found that SBTC is most often at C4 in women and C4/5 in men, IBTC is most often at C5 in women and men, and IBCC is most often at C6 in women and C6 or C6/7 in men in Chinese (Table 6). The results of our work are generally in good agreement with previous studies [1, 9, 11]. Contemporary anatomy texts indicate that the upper border of the thyroid cartilage is at C3/4 or C4 in the USA [11]. A more recent CT study of over 52 asymptomatic New Zealanders found that the vertebral level corresponding to the upper border of the thyroid cartilage was sexually dimorphic and was most often at C4 in women and C5 in men. The inferior border of the cricoid cartilage was also significantly different in women and men (C6 and C7, respectively) [9]. Additionally, we identified changes in these parameters with age, weight, height, and BMI (Table 5). We found that V1, V2, and V3 all increased with age. This may be due to the development and calcification of thyroid cartilage [12, 13]. However, it is curious that a higher BMI was correlated to an increase in D1, D2 and D3 but not correlated to V1, V2, and V3. We were also able to show that weight and height were correlated to increasing D1, D2, D3 and V1, V2, V3. This finding is not surprising given the increase in bone size with height and weight in the normal BMI range. However, the correlation coefficient between D1, D2, D3, and weight and height is higher than that between V1, V2, V3, and weight and height.

There is general agreement on the vertebral levels of key palpable landmarks in anterior cervical spine surgery. However, inaccurate preoperative skin positioning may lead to an enlarged intraoperative incision and range of exposure, resulting in an increase in both the operation time and tension of the intraoperative soft tissue traction [1]. Therefore, an appropriate skin incision can maximize the surgical field and avoid expanding the incision, which may otherwise affect the esthetics and increase the extent of the stripping, thus leading to an increased risk of postoperative anterior cervical hematoma and dysphagia due to elevated hemorrhage and continuous traction [3]. Kirschner wire and other fine metals are extensively used as a reference prior to skin cutting and decompression in anterior cervical approach surgery followed by C-arm perspective positioning. However, an acquaintance with the related anatomical positioning markers close to the cervical level contributes to a reduction in repeated perspective adjustments, thus saving a great deal of time and avoiding an increase in radiation exposure.

Preoperative positioning in anterior cervical approach surgery generally utilizes anatomical structures such as the angle of the mandible, hyoid bone, thyroid cartilage, cricoid cartilage, and carotid artery nodule [1, 6]. The mandibular angle is located at the interval between C2 and C3 [1], while the majority of cervical decompression surgeries are located below the C4 level [7]. Therefore, the angle of the mandible is not suitable, although the distance from it to the surgical level can be measured on preoperative X-ray films. The thyroid cartilage, however, is mostly located at the C4–C5 level according to the present study and is more or less fixed in front of the cervical vertebrae apart from when swallowing or intonation occurs. The cartilage does not easily move with body position but is easily accessible compared with the hyoid bone and carotid artery nodule. Additionally, the anatomical location of the thyroid cartilage angle is more obvious in men due to the presence of the Adam’s apple. Therefore, preoperative X-rays allow for the location of almost all anterior cervical approach surgical levels. Therefore, the corresponding surgical level can be judged rapidly based on the position of the SBTC, IBTC, and IBCC prior to skin cutting and decompression.

The thyroid cartilage or trachea and the posterior pharyngeal wall or esophagus in front of the surgical level should be pulled to one side during surgery. Therefore, a wide thyroid cartilage will often add to the difficulties incurred in exposing the surgical level. The thyroid cartilage is an important barrier in anterior cervical approach surgery. Chio et al. [4] discovered through intraoperative ultrasound positioning that the thyroid cartilage rarely rotated along with the traction of the retractor, while translation of the thyroid cartilage with the posterior pharyngoesophagus was common. The traction of the retractor should be increased when pulling a larger thyroid cartilage to sufficiently expose the surgical level. Nevertheless, increasing intraoperative traction may lead to cervical hematoma and soft tissue swelling. However, Han et al. [5] conducted a study in 2015 in which the tension of the retractor at the time of pulling and the pressure in the esophagus were measured. Moreover, it was verified that increasing the tension outside the thyroid cartilage would not increase the pressure in the esophagus. In contrast, translation of a larger thyroid cartilage with the posterior pharyngoesophagus can be seen at the time of traction, which can protect the PE wall [4].

The exact pathogenesis of cervical esophageal injury remains unknown [7]. Although the incidence of PE injury has steadily increased [14–16], PE injury is unfortunately hard to diagnose even with advancements in radiography [17]. Thus, the prevention of injury is most important during anterior cervical spine surgery. Direct injury with an instrument or retractor during dissection is a possible cause of PE injury [15, 18, 19]. Our results (Tables 3 and 4) suggest that the average transverse diameter of the SBTC and IBTC or IVCC gradually decreased in healthy adults from top to bottom, while that of the corresponding cervical vertebral body or intervertebral disc gradually increased. Therefore, the traction of the retractor should be increased when conducting high-level anterior cervical approach surgery to sufficiently expose the surgical level, which will therefore lead to cervical hematoma formation and soft-tissue swelling [3]. In contrast, the required traction of the retractor when performing lower cervical surgery is small since the coverage width of the thyroid cartilage gradually decreases; however, this may lead to direct pressure being placed on the esophagus. According to previous studies [15, 16], PE wall injury most often appears in lower cervical surgery (C5–C7), which may be related to a susceptibility to direct intraoperative traction [18, 19]. The anatomical location of the thyroid cartilage was further identified in this study, and it was found that the thyroid cartilage was mostly located above the C5 level (66.8%), while the trachea was mostly located below the C5 level. Such an anatomical feature provides a basis for the view that the thyroid cartilage does not protect the PE wall in lower cervical surgery (C5–C7). Therefore, when operating below the C5, special care should be taken during retraction to prevent injury of the esophagus wall.

This study does, however, acknowledge some weaknesses. Since this is a retrospective study, it is not possible to evaluate the patient face to face. Our sample size is considerable compared to other similar studies [8, 9]. The selection of individuals was only based on patient complaints and history records, and it is possible for these data to be skewed by outliers. A second limitation of our study is the relatively high proportion of elderly individuals enrolled. This occurred because age was not used as a screening criterion when cases were selected. While the aged-predominant nature of the population might limit our ability to draw conclusions about age variability, we do not believe it will limit the relevance of our work to anterior cervical spine surgery. Standardized head positions in CT scans are also different among different studies [9], but we have excluded patients with CT scan planes that are not consistent with the measurement plane because of incorrect head positions. Finally, it is important to acknowledge that the population in this study is drawn from an urban, university hospital in East China. Surgeons whose practice setting is significantly different from ours must be aware of the potential for ethnic- and race-based differences in normative values.

## Conclusions

In summary, we present normative values for the thyroid cartilage and cricoid cartilage at three planes of SBTC, IBTC, and IBCC in Chinese individuals. The individual and gender differences in the location of the thyroid cartilage and the size of the thyroid cartilage and the cricoid cartilage provided an anatomical basis to help localize the skin incision, to predict the difficulty of intraoperative exposure and retractor pulling, and to verify that the thyroid cartilage protects the PE wall.

## Abbreviations

AC: Anterior cervical
ACDF: Anterior cervical discectomy and fusion
D1, D2, D3: Transverse diameter of thyroid cartilage or trachea of SBTC, IBTC, and IBCC
IBCC: Inferior border of the cricoid cartilage
IBTC: Inferior border of the thyroid cartilage
ICC: Intra-class correlation coefficient
PBTC: Posterior border of thyroid cartilage
PE: Pharyngoesophageal
SBTC: Superior border of the thyroid cartilage
V1, V2, V3: Transverse diameter of vertebral body or intervertebral disc of SBTC, IBTC, and IBCC
